# Trajectory and early predictors of apathy development in first-episode psychosis and healthy controls: a 10-year follow-up study

**DOI:** 10.1007/s00406-020-01112-3

**Published:** 2020-03-04

**Authors:** Siv Hege Lyngstad, Erlend Strand Gardsjord, Magnus Johan Engen, Beathe Haatveit, Henrik Myhre Ihler, Kirsten Wedervang-Resell, Carmen Simonsen, Ingrid Melle, Ann Færden

**Affiliations:** 1 NORMENT, Division of Mental Health and Addiction, Oslo University Hospital & Institute of Clinical Medicine, University of Oslo, Oslo, Norway; 2 Division of Mental Health and Addiction, Nydalen DPS, Oslo University Hospital, Oslo, Norway; 3 Department of Psychiatric Research and Development, Division of Mental Health and Addiction, Oslo University Hospital, Oslo, Norway; 4 Division of Mental Health and Addiction, Early Intervention in Psychosis Advisory Unit for South East Norway, Oslo University Hospital, Oslo, Norway; 5 Division of Mental Health and Addiction, Department of Acute Psychiatry, Oslo University Hospital, Oslo, Norway

**Keywords:** First-episode psychosis, Follow-up, Course, Negative symptoms, Apathy, Avolition

## Abstract

Apathy is prevalent in first-episode psychosis (FEP) and associated with reduced global functioning. Investigations of the trajectory of apathy and its early predictors are needed to develop new treatment interventions. We here measured the levels of apathy over the first 10 years of treatment in FEP and in healthy controls (HC). We recruited 198 HC and 198 FEP participants. We measured apathy with the Apathy Evaluation Scale, self-report version, psychotic symptoms with the Positive and Negative Syndrome Scale, depression with the Calgary Depression Scale for Schizophrenia, functioning with the Global Assessment of Functioning Scale, and also estimated the duration of untreated psychosis (DUP). The longitudinal development of apathy and its predictors were explored using linear mixed models analyses. Associations to functioning at 10 years were investigated using multiple hierarchical linear regression analyses. In HC, mean apathy levels were low and stable. In FEP, apathy levels decreased significantly during the first year of treatment, followed by long-term stability. High individual levels of apathy at baseline were associated with higher apathy levels during the follow-up. Long DUP and high baseline levels of depression predicted higher apathy levels at follow-ups. The effect of DUP was persistent, while the effect of baseline depression decreased over time. At 10 years, apathy was statistically significantly associated with reduced functioning. The early phase of the disorder may be critical to the development of apathy in FEP.

**Electronic supplementary material:**

The online version of this article (10.1007/s00406-020-01112-3) contains supplementary material, which is available to authorized users.

## Introduction

Negative symptoms are core features of schizophrenia spectrum disorders and recognized as markers of an unfavorable illness course and outcome [1]. The etiology and pathogenesis of negative symptoms are mostly unknown, and current available treatments are not sufficient [2–4]. Negative symptoms are traditionally seen as stable; however, more recent follow-up studies indicate both symptom persistence and significant fluctuations [5–8]. Some studies indicate that the most noticeable changes occur during the first year of follow-up [9, 10], supporting the notion of a “critical period” of symptom evolvement [11]. However, the current evidence for a critical period for negative symptom evolvement is inconclusive [12].

Recent research indicates that negative symptoms comprise five sub-symptoms, clustering into two domains with different associations to the outcome: The expressive domain (i.e. blunted affect and alogia) and the experiential domain (i.e. anhedonia, avolition-apathy and asociality) [13–15]. These domains appear to have a continuous distribution that includes the general population [16]. There is evidence that avolition-apathy (“apathy” for short) is more strongly associated with a poor functional outcome than the other sub-symptoms [1, 17]. Apathy is usually defined as a reduction in goal-directed behavior due to a lack of motivation [18]. The prevalence of apathy in the early stages of a psychotic disorder can exceed 50% [19, 20], and higher levels are associated with male gender, reduced premorbid functioning, a long duration of untreated psychosis (DUP) and a diagnosis of schizophrenia [15, 19, 21, 22].

Despite the high prevalence of apathy in early psychosis, most studies have included participants with chronic illness [23–25], applied cross-sectional or short-term follow-up designs [21, 26] and/or used psychometric tools not primarily made to assess apathy [23, 27, 28]. The long-term development of apathy from the first treatment and its predictors thus remain mostly unexplored [29]. The only study so far investigating longer-term apathy development in first-episode psychosis (FEP) is the TIPS study [30]. At 10-year follow-up (10YFU), the study used a specialized psychometric tool to assess apathy, the Apathy Evaluation Scale-self-report version (AES-S) [31] and found that 30% of participants had high apathy levels, as defined by the AES-S. Using items from the Positive and Negative Syndrome Scale (PANSS) [32] as a proxy for AES measures, the trajectories of apathy over the follow-up period were then investigated retrospectively, with findings of a reduction in apathy levels during the first 1-to-2 years of treatment and stable levels from that point onward. No baseline variables predicted apathy levels at 10 years, but the use of different measures at different time-points limits interpretation. The study also lacked a healthy control group to examine the development of apathy over time.

The main aim of the current study was thus to investigate the development of apathy prospectively over 10 years in a FEP sample, using the AES at all time-points and additionally including a healthy control group (HC). Our research questions were:

1. How does apathy develop over 10 years in FEP compared to HC?
2. Do early clinical or demographic characteristics predict the development of apathy in FEP?
3. How prevalent is clinically significant apathy at 10YFU?
4. What are the functional consequences of high apathy levels at 10YFU?

We hypothesized that apathy would be higher in the FEP population than in HC, be predominantly stable over the follow-up period with changes primarily taking place early on. We also hypothesized that premorbid functioning and DUP would predict baseline levels of apathy and that premorbid function, DUP and baseline levels of apathy would predict the development of apathy over time. Finally, we hypothesized that the level of apathy would be a significant contributor to reduced functioning at 10YFU.

## Methods

### Participants

Two-hundred and fourteen participants with FEP aged 18 to 65 years were consecutively recruited from outpatient or inpatient units of hospitals in the regions of Oslo and Innlandet, as part of the Thematically Organized Psychosis (TOP) study in Norway. Inclusions into the study took place between March 2004 and December 2007 in Oslo, and between December 2007 and October 2009 at Innlandet. Participants were reassessed after 7 years (7YFU) at Innlandet, and after 10 years in Oslo (10YFU). A subset of the Oslo participants also had an intermediate assessment (6 and/or 12 months).

All FEP participants met the diagnostic criteria of a non-affective psychotic disorder, i.e. schizophrenia, schizophreniform disorder, schizoaffective disorder (“Schizophrenia spectrum disorders”) or delusional disorder, brief psychotic disorder or psychosis not otherwise specified. A psychotic episode was defined as having a score of ≥ 4 on items p1 (delusions), p2 (conceptual disorganisation), p3 (hallucinatory behavior), p5 (grandiosity), p6 (suspiciousness/persecution) or g9 (unusual thought content) for ≥ 1 week on the PANSS. Participants were not defined as FEP if they had previously received adequate treatment for psychosis (i.e. hospitalization or antipsychotic medication in adequate dosage for ≥ 12 weeks or until remission). Since some patients were not able to give informed consent during the acute phase, FEP participants were eligible for inclusion within 52 weeks of the start of first adequate treatment.

Exclusion criteria were: Not speaking a Scandinavian language, IQ < 70, current neurological or medical condition which could cause negative symptoms or psychosis, psychosis due to substance use, moderate/severe head injury prior to inclusion or during the follow-up period.

**Fig. 1:**
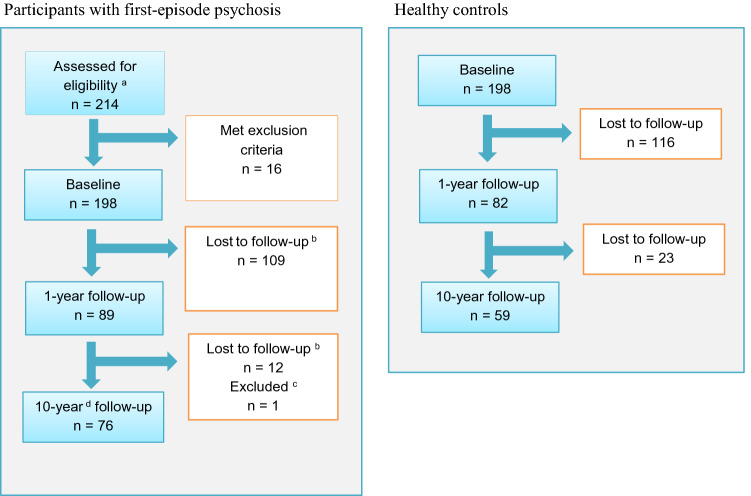
Participation in a 10-year follow-up of people with first-episode psychosis and in healthy controls. ^a^Patients with first-episode psychosis were consecutively referred to the study from their clinical units. Since Norwegian law does not allow researchers to access medical charts of patients before they give an informed consent or to keep data on those who do not consent, we have no report of the number of eligible patients that were not referred or said no to study referral. ^b^Nine participants had died (all at the Oslo Site), nine had moved abroad, 43 were untraceable, and 60 refused further participation. ^c^One participant was excluded due to a newly acquired severe head injury between 1 and 10 years. ^d^At Innlandet, mean follow-up time was 7.1 years

Based on these criteria, 16 participants initially deemed eligible were excluded, leaving 198 for analyses at baseline (BL) (Fig. 1). Of these, 98 (49%) had an intermediate assessment at 6MFU and/or at 1YFU. A total of 77 (41%) completed assessments at the long-term follow-up. One participant was excluded due to a severe head injury between 1 and 10YFU, leaving 76. Of the 121 lost to follow up, nine had died (all from Oslo), nine had moved abroad, and 43 were untraceable despite multiple attempts to contact them, and 60 said no to further participation.

The HC were 18–65 years old and were randomly selected from the national population registry of Norway [33], and invited to participate by letter. All HC were interviewed with the Primary Screening for Mental Disorders [34] at BL and follow-ups to ensure that they, or any first-degree relative, did not have a current or previous severe mental illness. The same exclusion criteria used for participants with FEP were applied, and 199 HC were included. One HC developed a severe mental illness during the follow-up and was excluded from analyses at both BL and follow-ups, leaving 198 HC at BL, 82 (41%) with intermediate measures (1YFU) and 59 (30%) at 10YFU (Fig. 1).

### Clinical assessment

At each follow-up point, participants were interviewed by psychologists or medical doctors, applying a comprehensive clinical assessment protocol. The Structured Clinical Interview for Mental Disorders (SCID-I) was used to diagnose participants, according to the DSM IV [35]. All interviewers completed a SCID-assessment training program based in the University of California, Los Angeles [36]. Diagnostic consensus meetings led by experienced clinical researchers were held regularly, and inter-rater reliability was found satisfactory [37]. Medical charts from in- and outpatient treatments during the follow-up were inspected to supplement information given by the participants.

Premorbid functioning was measured with the Premorbid Adjustment Scale (PAS) [38]. Since the baseline assessments were done in the mid-2000s, the structured interview for PAS published in 2009 was not used [39]. Scores were divided into age intervals (childhood ≤ 11 years, early adolescence 12–15 years, late adolescence 16–18 years, adult > 18 years), and further into social and academic functioning within each interval. To reduce chances of prodromal symptoms influencing adjustment, we only used childhood scores in the analyses, and PAS scores for patients with age at onset less than 12 years of age were treated as missing. The age at onset (AAO) refers to the individual’s age when the first psychotic episode started. Duration of untreated psychosis (DUP) was defined as the time in weeks from the first psychotic episode until first adequate treatment [40].

Psychotic and other symptoms were measured with the Positive and Negative Syndrome Scale (PANSS), divided into five factors (positive, negative, disorganized, depressed and excited) [41]. The Apathy Evaluation Scale self-report version (AES-S) was used to assess apathy [31]. A shortened 12-item version with superior psychometric properties in FEP was applied [42]. The AES-S has shown a high concordance with the clinician-rated version, AES-C, in a partly overlapping sample [43], and reliably distinguishes patients from HC [19, 25]. The AES-S maps one’s interests and engagement during the last month. A higher score indicates higher levels of apathy. Following previous studies [19, 30], we used a sum-score cut-off of ≥ 27 points (two standard deviations above mean for HC) to indicate clinically significant apathy. To better distinguish between apathy as a part of negative symptoms and symptoms of depression [44], depression was measured with the Calgary Depression Scale for Schizophrenia (CDSS) [45]. Higher CDSS scores indicate higher levels of depressive symptoms.

Global functioning was measured with the Global Assessment of Functioning Scale-split version, functioning subscale (GAF-F) [46]. Scores range from 0 (extremely impaired) to 100 (perfect function). Alcohol and drug use the last year were measured with the Alcohol Use Disorder Identification Test (AUDIT) [47] and the Drug Use Disorder Identification Test (DUDIT) [48], respectively.

The current load of antipsychotic medication (AP), were represented by dividing the actual daily dosage of used antipsychotics with its Defined Daily Dosage (DDD) (dosage recommended by the WHO Collaborating Centre for Drug Statistics Method [49]). If a participant used two or three different AP, one ratio was computed for each AP, and the ratios subsequently summarized to ‘Sum AP’.

### Statistical analyses

Analyses were carried out in the SPSS version 25. Variables were inspected for outliers, normality, collinearity and heteroscedasticity. Tests were two-tailed, and significance levels pre-set to 0.05.

#### Site characteristics and follow-up intervals

Mean long-term follow-up time was 7.1 years at Innlandet and 10.8 years in Oslo. We expected higher stability of symptoms and functioning this late in the course of illness and thus assumed that the difference in follow-up time would not have a significant influence on the results of the analyses. The ‘10YFU’ variables thus included measures from both 7 (Innlandet) and 10 years (Oslo). There could, however, be other systematic or random site differences. In our sample, patients from the rural communities at Innlandet had a longer DUP than Oslo (median DUP_Innlandet_ = 104 weeks; median DUP_Oslo_ = 52 weeks, *t *= − 4.4, *p* < 0.001) and a significantly higher proportion meeting a schizophrenia spectrum diagnosis at baseline (*χ*^2^ = 4.0, *p* = 0.045). ‘Inclusion site’ was thus adjusted for in the multivariate analyses in the case of a significant bivariate association between “Inclusion site” and other covariates and/or outcome variables in initial analyses.

#### Missing data

We evaluated differences in BL characteristics between those who completed and those who did not complete the long-term follow-up using *χ*^2^ test for categorical and t-tests or Mann–Whitney *U*-tests for continuous data (Table 5). Participants with a lower PANSS general symptoms score, male gender or non-European ethnicity were significantly less likely to complete the long-term follow-up assessments. No other significant differences were found.

The AES-S had no missing data in those who completed reassessment at each follow-up point. The GAF-F score was missing in one participant at 10YFU. For the CDSS, AUDIT and DUDIT, between one and five participants had missing scores for two or fewer items at one or more follow-up points. These missing items were replaced with item scores imputed as the mean value of the non-missing items for the scale in question for that participant at that specific follow-up. If more than two items were missing, which was the case in less than five participants, no imputations were done and the variable was treated as missing. Missing data did not exceed 4% for any BL data, except for the Sum AP, which had 7% missing.

#### Analyses

FEP and HC samples were analyzed separately for the first research question. We used a scatter-dot with a fitted regression line to explore the longitudinal development of apathy. To account for missing data and dependencies caused by repeated measurements, we then applied linear mixed models analyses [50]. In FEP, AES-S scores at four follow-up points were used as the dependent, continuous apathy variable. Longitudinal apathy development was described by employing a growth model, and maximum likelihood used to select the best-fitted model. Time was first introduced as fixed factor. We then explored whether a curvilinear function (time*time) improved model fit. Subsequently, random intercept and random slope were introduced, and an autoregressive heterogeneous (AR1H) covariance structure between them was inspected. The same procedure was then applied for HC separately, using the available three assessment points for the dependent, continuous apathy variable.

For the second research question, relevant early predictors and covariates of apathy development in FEP were chosen based on previous research and theory. We used Pearson’s bivariate correlation analyses to investigate associations between predictors, covariates and the AES-S scores at BL and 10YFU*.* Variables with significant (*p* ≤ 0.1) bivariate associations to apathy development were introduced into the linear mixed models analyses in order of lifetime appearance. Interaction effects with time were explored only for BL predictors with a significant association to apathy development. Such interaction effects describe whether the predictor’s effect on apathy development increases or decreases with time. Predictors and covariates with non-significant estimates (*p* > 0.05) were removed from the final equation. The following equation describes the basic model:

$$Y_{{{\text{ij}}}} = \, \left( {\beta_{0} + {\text{ b}}_{{0{\text{i}}}} } \right) \, + \, \left( {\beta_{{{\text{1ij}}}} + {\text{ b}}_{{{\text{1ij}}}} } \right)*{\text{time }} + \, \beta_{{{\text{2ij}}}} *{\text{time}}*{\text{time }} + \, \beta_{{{\text{3ij}}}} *{\text{predictor }} + \beta_{{{\text{4ij}}}} *{\text{predictor}}*{\text{time }} + \, \varepsilon_{{{\text{ij}}}}$$

*Y*_*ij*_ is apathy in an individual *i* = 1…, 198 at year *j* = 1…, 10*.* *β*_0_…*β*_4ij_ are the estimates of the population’s means (i.e. fixed effects). The *b*_0i_ and *b*_1ij_ represent the specific random variation between individuals in BL apathy levels and in the slope of apathy development, respectively.

For the third research question, we employed Pearson’s bivariate correlation analyses to evaluate the association between GAF-F at 10YFU and concurrent symptoms, diagnosis and demographic variables in FEP. Multiple hierarchical linear regression analyses were used to investigate associations to GAF-F further. Independent variables with significant (*p* ≤ 0.1) bivariate associations to GAF-F were introduced in a block-wise manner, with the AES-S score in the final block.

## Results

Table 1 displays the characteristics of HC and participants with FEP. A total of 198 FEP patients and 198 HC were included at BL. At 1YFU, 89 patients and 82 HC were reassessed, while 76 of the included patients and 52 of the HC were reassessed at 10YFU. At BL, 36% of the FEP patients and 48% of the HC were female. The mean age in FEP and HC was 27 years and 33 years, respectively. Among patients, 67% had schizophrenia, schizophreniform or schizoaffective disorder diagnosis (i.e. a schizophrenia spectrum disorder).

**Table 1:** Characteristics of first-episode psychosis participants and healthy controls during follow-up

	Baseline		6MFU		1YFU		10YFU	
	FEP	HC	FEP	HC	FEP	HC	FEP	HC
N (%)	198	198	49 (24.7)	–	89 (44.9)	82 (41.4)	76 (40.7^e^)	59 (29.8)
Gender female (n/%)	72 (36.4)	94 (47.5)	24 (49.0)	–	35 (39.3)	39 (47.6)	35 (46.1)	27 (45.8)
Age	27.2 (8.5)	32.6 (9.1)	28.2 (8.7)	–	27.6 (7.2)	–	35.9 (8.9)	39.9 (6.9)
Single (n/%)	146 (73.7)	–	–	–	–	–	41 (43.9)	12 (20.3)
Ethnicity European (n/%)	155 (78.3)	196 (99)	37 (75.5)	–	65 (73.0)	82 (100)	67 (88.2)	59 (100)
Working or studying (n/%)	71 (36.0)	–	–	–	–	–	59 (77.6)	–
IQ^a^	100.5 (13.8)	114.5 (9.5)	–	–	–	–	–	–
Premorbid functioning								
PAS social (median/range)	1.0 (0–6.0)	–	–	–	–	–	–	–
PAS acad. (median/range)	1.5 (0–5.5)	–	–	–	–	–	–	–
AAO psychosis	23.3 (8.1)	–	–	–	–	–	–	–
DUP weeks (median/range)	75 (1–1560)	–	–	–	–	–	–	–
Diagnosis (n/%)								
Schizophrenia spectrum^b^	134 (67.7)	–	–	–	–	–	58 (76.3)	–
Other psychosis ^c^	64 (32.3)	–	–	–	–	–	18 (23.7)	–
Symptoms and functioning								
PANSS positive	16.2 (5.0)	–	12.3 (4.5)	–	13.0 (5.1)	–	12.5 (5.0)	–
PANSS negative	15.5 (6.6)	–	14.7 (5.2)	–	13.5 (5.0)	–	12.2 (5.0)	–
PANSS general	34.0 (8.3)	–	27.8 (7.7)	–	27.3 (6.9)	–	26.5 (8.1)	–
AES-S	28.7 (7.6)	17.6 (4.2)	26.1 (7.5)	–	24.6 (7.0)	17.2 (4.0)	24.7 (7.1)	18.1 (4.5)
AES-S ≥ 27 (n/%)	118 (59.6)	8 (4.0)	25 (51.0)	–	31 (34.8)	2 (2.4)	28 (36.8)	3 (5.1)
CDSS	6.8 (4.9)	–	3.8 (4.5)	–	3.8 (3.4)	–	2.8 (3.1)	–
AUDIT (median/range)	5.0 (0–38)	–	4.0 (0–31)	–	4.0 (0–29)	5.0 (0–14)	4.0 (0–28)	5.0 (1–12)
DUDIT (median/range)	0.0 (0–44)	–	0.0 (0–32)	–	0.0 (0–34)	0.0 (0–10)	0.0 (0–42)	0.0 (0–5)
Sum AP^d^	0.9 (0.8)	–	–	–	1.1 (0.89)	–	1.3 (0.9)	–
GAF-F	42.6 (12.5)	–	55.0 (16.1)	–	53.3 (16.6)	–	58.4 (16.3)	–

Unless otherwise specified, values are given in means (standard deviation)

*6MFU* six-months follow-up, *1YFU* one-year follow-up, *10YFU* ten-year follow-up, *IQ* intelligence quotient, *AAO psychosis* age at onset of first psychotic episode, *PAS* premorbid assessment scale, *DUP* duration of untreated psychosis, *PANSS* positive and negative syndrome scale, *AES-S* apathy evaluation scale-self-report version, *CDSS* calgary depression scale for schizophrenia, *AUDIT* alcohol use disorder identification test, *DUDIT* drug use disorder identification test, *GAF-F* global assessment of functioning scale, split version, Functioning subscale, *Sum AP* weighted sum of antipsychotic medication

^a^The average IQ for HC in the present sample are parallel to the findings reported by the Knowledge Centre for the Health Services at The Norwegian Institute of Public Health, evaluating the psychometric properties of the Wechsler Abbreviated Scale of Intelligence (WASI) in Norwegian study samples [79]

^b^Schizophrenia spectrum = Schizophrenia, schizophreniform and schizoaffective disorders

^c^Other psychosis = Brief Psychotic Disorder, Delusional Disorder and Psychosis Not Otherwise Specified (PNOS)

^d^The actual daily dose used (of each antipsychotic medication) was divided by the defined daily dosage (DDD) for that specific preparation. These ratios (for a maximum of three simultaneously used antipsychotics) were then summed and called Sum AP, a proxy for the total antipsychotic load in each participant

^e^Of the 198 included at BL, nine had died and nine had moved abroad. At 10YFU, *n* = 77 were reassessed. One of these was excluded from analyses at 10YFU due to a severe head injury since 1YFU. Retention rate was estimated based on the 189 participants who were alive and available to follow-up

### Development of apathy in HC

**Fig. 2:**
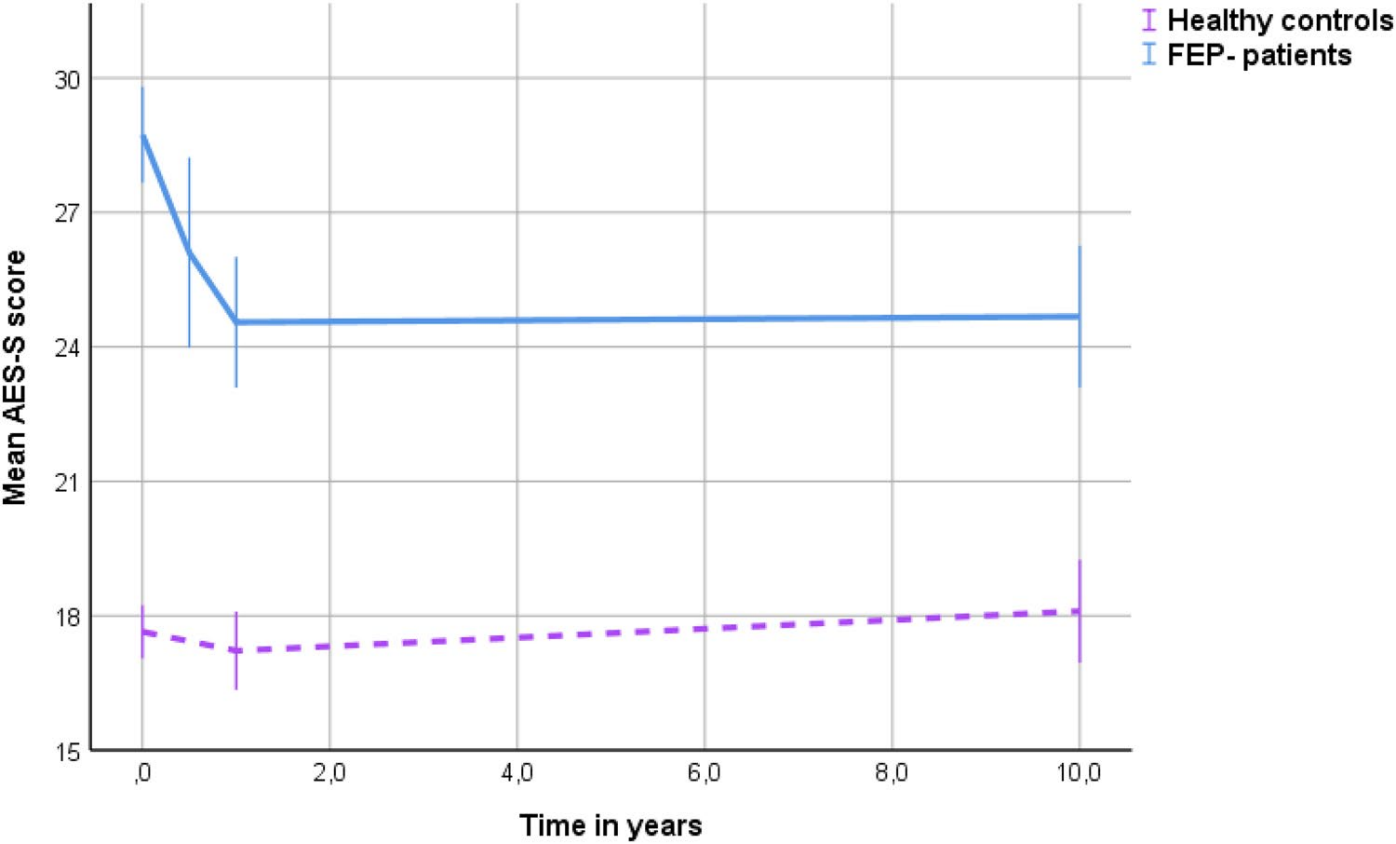
Development of apathy (AES-S scores) in first-episode psychosis (FEP) patients and in healthy controls during the 10-year follow-up

Development of apathy in HC is presented in Fig. 2. Mean apathy levels appeared stable over the follow-up period, as indicated by a non-significant fixed effect of time in the apathy growth model (*p* = 0.215). However, apathy levels varied significantly between individuals at BL and between individuals over time, as shown by a significant effect of a random intercept (*p* < 0.001) and random slope (*p* = 0.019), respectively. The individual level of apathy at BL was not associated with the individual development of time, as indicated by a non-significant covariance between the random intercept and slope (*p* = 0.106). Gender and age did not contribute significantly to the model.

### Development of apathy in participants with FEP

Apathy development in FEP participants is displayed in Fig. 2. The scatter-dot regression line indicated that apathy levels declined during the first year, levelling off thereafter. In the growth model, apathy levels decreased over the long-term follow-up, i.e. there was a significant, fixed effect of time (− 2 log likelihood = 2836.8; BIC = 2854.9, *p* = 0.002). When quadratic time (time*time) was added to the equation, the model fit was improved (− 2 log likelihood = 2818.8, BIC = 2842.9). The linear effect of time was negative, while the quadratic effect was positive (both: *p* < 0.001). Apathy levels significantly varied between individuals at BL, as indicated by a significant random intercept (*p* < 0.001). The random slope and the covariance between the random intercept and slope did not significantly improve model fit, which suggested that the development of apathy did not significantly differ between individuals over time, with an enduring effect of baseline apathy levels.

### Early clinical and demographic predictors of apathy development in FEP

Bivariate correlations are presented in Table 2, followed by the linear mixed models analyses in Table 3. The AES-S level at BL was significantly associated with the PAS social and academic scores, DUP, concurrent CDSS, and the AES-S and CDSS at 10YFU. The AES-S level at 10YFU was significantly associated with gender, DUP, concurrent CDSS and PANSS disorganized symptoms.

**Table 2:** Pearson’s bivariate correlation analyses between patient characteristics at baseline and 10 years, AES-S at baseline and 10 years and GAF-F at 10 years

Demographic and clinical variables	AES-S BL	AES-S 10Y	GAF-F 10Y
*N*	198	76	76
Inclusion site	0.22*	0.32**	− 0.07
Gender	0.00	− 0.24*	0.21
PAS social childhood^a^	0.19**	0.06	− 0.16
PAS acad. childhood^a^	0.14*	0.06	− 0.14
AAO psychosis	− 0.13	− 0.08	0.10
DUP^a^	0.19**	0.24*	− 0.32**
Schizophrenia spectrum BL^b^	0.10	0.04	− 0.34**
Schizophrenia spectrum 10Y^b^	0.11	0.03	− 0.36**
PANSS pos. BL	0.08	0.06	− 0.17
PANSS pos. 10Y^a^	0.11	0.18	− 0.56**
PANSS disorg. BL^a^	− 0.04	0.14	− 0.29*
PANSS disorg. 10Y^a^	0.05	0.39**	− 0.58**
AES-S BL	–	0.42**	− 0.16
AES-S 10Y	–	–	− 0.49**
PANSS insight BL (g12)	− 0.09	− 0.12	− 0.11
PANSS insight 10Y (g12)^a^	0.07	0.21	− 0.53**
CDSS BL^a^	0.44**	0.22	− 0.19
CDSS 10Y^a^	0.33**	0.59**	− 0.48**
AUDIT BL^a^	0.01	0.14	0.14
AUDIT 10Y^a^	− 0.16	0.10	0.02
DUDIT BL^a^	− 0.03	− 0.05	− 0.02
DUDIT 10Y^a^	− 0.03	− 0.08	− 0.09
Sum AP BL^a^	− 0.10	− 0.19	0.04
Sum AP 10Y^a^	− 0.10	− 0.06	− 0.18
GAF-F BL	− 0.26**	− 0.32**	0.39**

*BL* baseline, *FU* follow-up, *10Y* ten-year, *PAS* premorbid adjustment scale, *DUP* duration of untreated psychosis, *GAF-F* global assessment of functioning scale-function subscale, *PANSS* positive and negative syndrome scale, *AES-S* apathy evaluation scale-self report version, *CDSS* calgary depression scale for schizophrenia, *DUDIT* drug use disorder identification test, *AUDIT* alcohol use disorder identification test, *Sum AP* sum antipsychotic medication; the actual daily dose used (of each antipsychotic medication) was divided by the defined daily dosage (DDD) for that specific preparation. These ratios (for a maximum of three simultaneously used antipsychotics) were then summed and called Sum AP, representing the total antipsychotic load in each participant

^*^p < 0.05; **p < 0.01

^a^PAS social, DUP, CDSS 10Y, Sum AP BL, AUDIT and DUDIT (BL and 10Y) were log10-transformed, CDSS BL, PANSS insight 10Y and Sum AP 10Y were square root transformed due to skewness

^b^Schizophrenia spectrum = Schizophrenia, schizophreniform and schizoaffective disorders

**Table 3:** Linear mixed model analysis. Early predictors of apathy (AES-S) development in first-episode psychosis during 10-year follow-up

Parameter	Estimate	SE	*t*	*p *value	95% CI for *t*	
					Lower	Upper
Intercept	22.17	1.19	18.61	< 0.001	19.82	24.51
Time	− 2.78	0.77	− 3.63	< 0.001	− 4.29	− 1.27
Time*time	0.27	0.07	3.64	< 0.001	0.12	0.42
DUP ^a^	1.47	0.59	2.47	0.014	0.29	2.64
CDSS	0.59	0.10	6.07	< 0.001	0.40	0.78
CDSS*time	− 0.05	0.01	− 3.36	0.001	− 0.08	− 0.02

Estimate, SE, *t*, *p* and 95% CI refer to the numbers in the final model, adjusted for Inclusion site

Inclusion Site additionally showed a significant association with apathy development. Participants recruited at Innlandet had an increased likelihood of higher apathy levels during the follow-up (Est. = 2.15, *p* = 0.048)

*SE* standard error, *CI* confidence interval, time time in years from baseline to 10 years, *DUP* duration of untreated psychosis, *CDSS* calgary depression scale for schizophrenia

^a^DUP was log 10-transformed due to a severely skewed distribution

In the linear mixed models analysis, DUP had a significant, positive association with the development of apathy. There was an enduring effect of DUP, as shown by a non-significant interaction effect of DUP*time. Baseline CDSS levels showed a significant, positive association with the development of apathy. The interaction term CDSS*time was negative and statistically significant, indicating that the effect of BL depression decreased with time. Gender, AAO, PAS, BL disorganized symptoms, AUDIT, DUDIT, Sum AP, or having a schizophrenia spectrum diagnosis did not contribute significantly to the model. The inclusion site was, however, significantly associated with apathy development, with higher apathy scores at the Innlandet site, also after correcting for other statistically significant variables in the equation.

### Prevalence of clinically significant apathy at 10 years and the associations between apathy and global functioning

The prevalence of clinically significant apathy at 10YFU was 5% in HC and 37% in FEP participants (Table 1). Results from the multiple hierarchical linear regression analysis at 10YFU are shown in Table 4. Concurrent positive and disorganized symptoms, and having a schizophrenia spectrum diagnosis, had statistically significant, negative associations with GAF-F. After adjusting for these variables and concurrent depression, apathy added 5% to the explained variance in GAF-F. Age, gender, AUDIT, DUDIT and Sum AP did not contribute significantly to the model.

**Table 4:** Multiple hierarchical regression analyses at 10-year follow-up in first-episode psychosis, GAF-F^a^ is the dependent variable

	10Y follow-up variable	*b*	*Std. β*	*t*	95% CI for *β*	${R}^{2}$ change	${R}^{2}$^b^	*p* value
	Constant	101.38	–	17.53	(89.85, 112.92)	–	–	< 0.001
1st block	Schizophrenia spectrum	− 7.01	− 0.18	− 2.22	(−13.30, − 0.72)	0.126	0.126	0.030
2nd block	PANSS positive	− 1.33	− 0.33	− 3.61	(− 2.06, − 0.59)	–	–	0.001
	PANSS disorganized	− 1.90	− 0.25	− 2.73	(− 3.29, − 0.51)	0.346	0.472	0.008
3rd block	CDSS	− 0.41	− 0.08	− 0.78	(− 1.47, 0.65)	0.053	0.525	0.440
4th block	AES-S	− 0.67	− 0.29	− 2.85	(− 1.14, − 0.20)	0.050	0.575	0.006

*10Y* ten-year, *Schizophrenia spectrum* schizophrenia, schizoaffective and schizophreniform disorders, *PANSS* positive and negative syndrome scale, *CDSS* calgary depression scale for schizophrenia, *AES-S* Apathy Evaluation Scale—Self-report version

^a^Global Assessment of Function Scale, split version-functioning subscale

^b^Neither age, gender, alcohol use (AUDIT), drug use (DUDIT) nor the amount of antipsychotic medication (Sum AP) contributed significantly to the model. Adjusted R^2^ for the total model = 0.545

## Discussion

### Main findings

We found a significant decrease in mean apathy scores during the first year of treatment in FEP, followed by long-term stability over the next 6 to 9 years. A high BL apathy score increased the likelihood of apathy scores above the group mean throughout the follow-up. Also, a long DUP and high BL depression score predicted higher apathy scores over the follow-up period. However, while the effect of BL depression levels decreased over time, the effect of DUP persisted. The mean apathy scores in the HC group were lower and stable over time, but with inter-individual variation both in BL levels and in later trajectories. Accordingly, the BL apathy score was not equally predictive of the later development of apathy in HC.

In FEP, a schizophrenia spectrum diagnosis together with concurrent positive- and disorganized symptoms together were significantly associated with poorer global functioning at the long-term follow-up. The level of apathy had an independent and statistically significant influence on global functioning also after adjusting for other clinical characteristics in the multivariate analyses.

### Development of apathy in participants with FEP

The finding of an overall decrease in apathy levels in the long-term is in line with results from two previous follow-up studies from FEP [30] and first-admission schizophrenia participants [51]. In the FEP TIPS study, a group characterized by enduring high apathy levels was discernible in the second year of treatment. Another group with lower and decreasing apathy levels over time explained most of the overall reduction in apathy levels in the total sample [30]. The primary reduction in apathy levels both in the TIPS study and the current study took place within the first years of treatment. This finding supports that the notion of a critical period for symptom development in FEP, i.e. a time interval where symptoms may be more amenable to interventions, also comprises the development of apathy [11].

We also found that the individual variations in apathy levels already at BL were carried forward through the follow-up period, corresponding to the “persistently high apathy” group in the TIPS study [30]. Since the TIPS was an early intervention study, it recruited FEP with a short DUP during their first week of treatment [52], which may explain why symptom trajectories were less stable over the first years of treatment. Taken together this indicates that factors influencing apathy trajectories are in place well before the first adequate treatment of the psychotic illness. This notion is supported by findings of stable negative symptoms in ultra-high-risk populations [53].

### Early clinical or demographic predictors of apathy development in FEP

In line with our hypothesis and evidence from more broadly defined negative symptoms [54], we found that a long DUP in FEP predicted higher levels of apathy throughout the follow-up period. This finding expand on previous research from our group that identified statistically significant associations between a long DUP and high apathy scores at 1-year follow-up in a sample partly overlapping with the current [21]. The TIPS study found statistically significant associations between a long DUP and high negative symptoms in the short term. DUP did, however, not predict the level of apathy at 10 years [30], possibly because the short median DUP in the TIPS study reduced statistical power. We do not know by which mechanisms, DUP contributes to a poor outcome [55–57]. However, findings from the TIPS study indicate that shortening DUP will lead to lower levels of negative symptoms and improved functioning from treatment start through long-term follow-ups [58–61]. In our sample, patients from Innlandet had a longer DUP than Oslo. This may partly explain why Innlandet also had higher apathy scores at BL (mean AES-S Innlandet = 31.6 (7.5), mean AES-S Oslo = 27.7 (7.5), *t* = − 3.2, *p* = 0.002) and at 10YFU (mean AES-S Innlandet = 27.5 (7.2); mean AES-S Oslo = 22.9 (6.4), *t* = − 3.0, *p* = 0.004) [54].

The associations between BL depression and the development of apathy is intriguing. Depression is common also in non-affective psychotic disorders, especially in FEP [62, 63]. Although the phenomenology of depressive symptoms resembles those of negative symptoms [64], the different symptoms do not cluster together in factor analyses and show modest or inconsistent overlap in both cross-sectional- and longitudinal studies [65]. Research suggests that low mood and suicidal ideation are more linked to depressive symptoms and alogia/blunted affect more linked to negative symptoms, while reduced motivation (i.e. apathy) and anhedonia are common to both [66]. The association of apathy-anhedonia to both depression and negative symptoms indicates similarities in underlying CNS functions [67].

We found that the effect of BL depression on apathy trajectories decreased over time, while the cross-sectional association between concurrent depressive symptoms and apathy was stable. The results are in line with findings from a 13-year follow-up study of early psychosis, describing three trajectories for negative symptoms, where the high-and-increasing trajectory was predicted by BL depression, cognitive dysfunction and reduced premorbid functioning [68]. Another study of the longitudinal development of anhedonia/apathy and depressive symptoms in FEP found that the symptom domains levelled off after 2-to-5 years, while the associations between concurrent levels of apathy and depression increased in strength in the female participants over time [51]. Due to sample size and participant attrition, our findings should be interpreted with caution. They nevertheless serve as an argument for careful assessment- and active treatment of depression in FEP [69–71].

Finally, we expected that participants with poor premorbid adjustment as measured by the PAS and/or an earlier AAO had higher levels of apathy as a correlate of a more severe, neurodevelopmentally based illness. We did, however, not find any significant associations between PAS, AAO and apathy development in the multivariate analyses. We also hypothesized that a high BL Sum AP was associated with higher levels of apathy, since AP side effects may mimic negative symptoms [67]. Again, there were no significant associations between BL Sum AP and levels of apathy.

### Prevalence of clinically significant apathy at 10 years and associations to global functioning

In line with previous long-term studies in FEP [30], the prevalence of clinically significant apathy was substantial. Apathy also had an independent negative association with global functioning at 10 years. While the cross-sectional design for this particular research question precludes causal inference, our findings corroborate previous cross-sectional findings at BL and 1YFU in overlapping samples to the current sample [19, 21] and the TIPS study [30], and thus add to the suggested burden of apathy in psychosis [17].

## Strengths and limitations

The main strengths of this study include a richly phenotyped FEP sample and a prospective study design with a long follow-up period and a healthy control group. We also used validated psychometric tools, including a specialized tool for the assessment of apathy that was applied at BL and all follow-up assessments. Study participants were recruited through the Norwegian mental health care system, which is available to all citizens independent of socioeconomic status and thus increases the representativity of the study sample. Finally, we used robust statistical methods to handle dependencies in the data set.

There are also some limitations: First, we were not able to fully match FEP-participants with HC due to sample size. Second, our study may be subject to bias if reduced insight into illness impairs the ability to self-report apathy or the CDSS and AES-S do not adequately distinguish depression from apathy. However, recent research suggests that people with schizophrenia are aware of and report negative symptoms in a similar manner to external observers [22, 72], in contrast to findings from older studies [73, 74]. The AES-S shows a high concordance with the clinician-rated AES-C in FEP [43], and the PANSS insight item was not significantly associated with AES-S at BL or 10YFU. Additionally, the CDSS was designed to reduce confounding from negative symptoms [45].

Third, due to state-effects, depressed participants may evaluate themselves as more apathetic than others perceive them. Fourth, apathy was not assessed between years one and ten, and further variability in the trajectory may thus go unobserved.

Fifth, our sample size was limited, with subsequent attrition of participants. Our long-term attrition rate (59%) is at the same level as naturalistic FEP studies [75–77] but higher than in the TIPS (38%) [30] and OPUS cohorts (39%) [6], where retention can be boosted by the intervention designs or more frequent follow-ups. Attrition analyses revealed that being male, having non-European ethnicity or a lower BL PANSS general symptom score was associated with an increased likelihood of study drop-out at 10YFU. We did, however, not find any differences in other variables of interest, including DUP, BL AES-S, BL CDSS or BL GAF-F scores in follow-up analyses (Table 5). Follow-up analyses of BL symptoms, demographics and BL functioning across genders and ethnicity (data not shown), found no statistically significant differences in most variables of interest, including DUP, AES-S, the five PANSS factors and the GAF-F. Men were more likely to be single, have lower premorbid academic functioning, lower BL levels of depression and use more drugs. Europeans were more likely to use alcohol and have poorer premorbid social functioning, but higher IQ scores. We were not able to find a systematic trend of attrition that could affect our results and linear mixed models analysis is a recommended and robust statistical method when data are missing. Baseline predictors of attrition are regularly used to evaluate the likelihood of selection bias in longitudinal studies. The estimates of associations between variables are, however, not necessarily affected by attrition in long-term longitudinal studies, even in the presence of differences in the mean scores of BL variables between completers and non-completers [78]. We thus assume that the follow-up sample at 10YFU was likely to be representative for the general distribution of symptoms and functioning in our full FEP sample.

**Table 5:** Comparisons of baseline characteristics between completers and non-completers at 10-year follow-up

Baseline variable	Completers	Non-completers	Statistic (*X*^2^*, t, U*)	*p* value
*N*	77	121		
Gender (male)	33.3%	66.7%	*X*^*2*^ = 4.50	0.034
Age (median)	23.0	25.0	*U* = 4239.5	0.286
Single	38.4%	61.6%	*X*^*2*^ = 0.07	0.797
Non-European ethnicity	20.9%	79.1%	*X*^*2*^ = 7.45	0.006
Working	40.8%	59.2%	*X*^2^ = 0.18	0.673
Educational years	12.1	12.0	*t* = − 0.17	0.863
IQ	101.2	99.9	*t* = − 0.61	0.545
PAS social childh. (median)	1.3	1.0	*U* = 3950.0	0.246
PAS acad. childh. (median)	1.5	1.5	*U* = 4270.5	0.867
AAO psychosis	22.3	23.9	*t* = 1.31	0.190
DUP^a^	1.8	1.7	*t* = − 0.91	0.362
Schizophrenia spectrum^b^	41.8%	58.2%	*X*^2^ = 1.47	0.225
PANSS positive	16.7	15.9	*t* = − 1.21	0.229
PANSS negative (median)	15.0	14.0	*U* = 4611.0	0.901
PANSS general	35.5	33.0	*t* = − 2.10	0.037
AES-S	28.9	28.6	*t* = − 0.22	0.825
AES-S ≥ 27	40.7%	59.3%	*X*^2^ = 0.39	0.531
CDSS	7.1	6.6	*t* = − 0.69	0.490
AUDIT (median)	6.0	5.0	*U* = 3784.5	0.142
DUDIT (median)	0.0	0.0	*U* = 4150.5	0.328
Sum AP (median)	0.7	1.0	*U* = 3683.0	0.377
GAF-F	42.2	42.8	*t* = 0.346	0.730

*IQ* intelligence quotient, *PAS* premorbid assessment scale, *AAO psychosis* age at onset of first psychotic episode, *DUP* duration of untreated psychosis, *PANSS* positive and negative syndrome scale, *AES-S* apathy evaluation scale-self-report version, *CDSS* calgary depression scale for schizophrenia, *AUDIT* alcohol use disorder identification test, *DUDIT* drug use disorder identification test, *Sum AP* weighted sum of antipsychotic medication, *GAF-F* global assessment of functioning scale, split version, Functioning subscale

^a^DUP was log10-transformed due to skewness

^b^Schizophrenia spectrum = Schizophrenia, Schizoaffective and Schizophreniform disorders

Sixth, to ensure that also initially acutely psychotic participants were able to give informed, written consent, FEP patients were eligible to enter the study up to 52 weeks after the start of the first adequate treatment, which could introduce more heterogeneity in BL symptom scores. Both positive- and depressive symptoms are causes of secondary negative symptoms and are higher at the start of the first adequate treatment. The observed decline from BL to 1YFU in levels of apathy may thus have been higher if the whole sample had entered the study at the start of the first adequate treatment.

## Conclusion and clinical implications

The current study supports the notion that the early treated- and untreated phases of the first psychotic episode is a critical period for the development of apathy. Based on the long-term effects of DUP, we can hypothesize that detecting and treating psychosis adequately at an early stage could reduce long-term apathy levels. The effect of BL depression on early apathy levels supports the idea of more active treatment of depression in FEP [69–71]. Considering the lack of evidence-based treatments for negative symptoms, efforts to reduce DUP and to treat co-occurring depressive symptoms could help to prevent high levels of apathy in the long term and thus improve functional outcome.

## Electronic supplementary material

Below is the link to the electronic supplementary material.

Supplementary file1 (PDF 208 kb)
