# Supplementary material for: Trajectory and early predictors of apathy development in first-episode psychosis and healthy controls: a 10-year follow-up study
Source: Eur Arch Psychiatry Clin Neurosci. 2020 Mar 4;270(6):709–22. doi: 10.1007/s00406-020-01112-3 (PMC7423800; doi:10.1007/s00406-020-01112-3)
Supplement: Supplementary file 1 — Supplementary file1 (PDF 208 kb) [file 406_2020_1112_MOESM1_ESM.pdf]

* Encoding: UTF-8.

****

*1. The growthmodel:

*Fixed effect of time:

```
DATASET ACTIVATE DataSet1.

MIXED Apathy_development WITH Time_years

  /CRITERIA=CIN(95) MXITER(100) MXSTEP(10) SCORING(1) SINGULAR(0.000000000001)
HCONVERGE(0,

    ABSOLUTE) LCONVERGE(0, ABSOLUTE) PCONVERGE(0.000001, ABSOLUTE)

  /FIXED=Time_years | SSTYPE(3)

  /METHOD=ML

  /PRINT=SOLUTION TESTCOV.
```

*Fixed effect of time plus time*time

```
MIXED Apathy_development WITH Time_years

  /CRITERIA=CIN(95) MXITER(100) MXSTEP(10) SCORING(1) SINGULAR(0.000000000001)
HCONVERGE(0,

    ABSOLUTE) LCONVERGE(0, ABSOLUTE) PCONVERGE(0.000001, ABSOLUTE)

  /FIXED=Time_years Time_years*Time_years | SSTYPE(3)

  /METHOD=ML

  /PRINT=SOLUTION TESTCOV.
```

*Fixed effects of time, time*time plus a random intercept:

```
MIXED Apathy_development WITH Time_years
  /CRITERIA=CIN(95) MXITER(100) MXSTEP(10) SCORING(1) SINGULAR(0.000000000001)
HCONVERGE(0,
    ABSOLUTE) LCONVERGE(0, ABSOLUTE) PCONVERGE(0.000001, ABSOLUTE)
  /FIXED=Time_years Time_years*Time_years | SSTYPE(3)
  /METHOD=ML
  /PRINT=SOLUTION TESTCOV
  /RANDOM=INTERCEPT | SUBJECT(id) COVTYPE(VC).
```

*Adding the random effect of time to the model above:

```
MIXED Apathy_development WITH Time_years
  /CRITERIA=CIN(95) MXITER(100) MXSTEP(10) SCORING(1) SINGULAR(0.000000000001)
HCONVERGE(0,
    ABSOLUTE) LCONVERGE(0, ABSOLUTE) PCONVERGE(0.000001, ABSOLUTE)
  /FIXED=Time_years Time_years*Time_years | SSTYPE(3)
  /METHOD=ML
  /PRINT=SOLUTION TESTCOV
  /RANDOM=INTERCEPT Time_years | SUBJECT(id) COVTYPE(VC).
```

*Inspecting the AR1Heterogeneous covariance between random intercept and random slope:

```
MIXED Apathy_development WITH Time_years
  /CRITERIA=CIN(95) MXITER(100) MXSTEP(10) SCORING(1) SINGULAR(0.000000000001)
HCONVERGE(0,
    ABSOLUTE) LCONVERGE(0, ABSOLUTE) PCONVERGE(0.000001, ABSOLUTE)
  /FIXED=Time_years Time_years*Time_years | SSTYPE(3)
  /METHOD=ML
  /PRINT=SOLUTION TESTCOV
  /RANDOM=INTERCEPT Time_years | SUBJECT(id) COVTYPE(ARH1).
```

****

*2. Bivariate associations between the apathy development and relevant early predictors from the correlation analyses; exploring interaction-effects with time for significant (p<0.1) predictors only:

MIXED Apathy_development WITH Time_years Gender_new

/CRITERIA=CIN(95) MXITER(100) MXSTEP(10) SCORING(1) SINGULAR(0.000000000001) HCONVERGE(0,

ABSOLUTE) LCONVERGE(0, ABSOLUTE) PCONVERGE(0.000001, ABSOLUTE)

/FIXED=Time_years Time_years*Time_years Gender_new | SSTYPE(3)

/METHOD=ML

/PRINT=SOLUTION TESTCOV

/RANDOM=INTERCEPT | SUBJECT(id) COVTYPE(VC).

MIXED Apathy_development WITH Time_years Age

/CRITERIA=CIN(95) MXITER(100) MXSTEP(10) SCORING(1) SINGULAR(0.000000000001) HCONVERGE(0,

ABSOLUTE) LCONVERGE(0, ABSOLUTE) PCONVERGE(0.000001, ABSOLUTE)

/FIXED=Time_years Time_years*Time_years Age | SSTYPE(3)

/METHOD=ML

/PRINT=SOLUTION TESTCOV

/RANDOM=INTERCEPT | SUBJECT(id) COVTYPE(VC).

MIXED Apathy_development WITH Time_years Inclusion_Site_BL_patients

/CRITERIA=CIN(95) MXITER(100) MXSTEP(10) SCORING(1) SINGULAR(0.000000000001) HCONVERGE(0,

ABSOLUTE) LCONVERGE(0, ABSOLUTE) PCONVERGE(0.000001, ABSOLUTE)

/FIXED=Time_years Time_years*Time_years Inclusion_Site_BL_patients | SSTYPE(3)

/METHOD=ML

/PRINT=SOLUTION TESTCOV

```
  /RANDOM=INTERCEPT | SUBJECT(id) COVTYPE(VC).

MIXED Apathy_development WITH Time_years sosvenb0
  /CRITERIA=CIN(95) MXITER(100) MXSTEP(10) SCORING(1) SINGULAR(0.000000000001) HCONVERGE(0,
    ABSOLUTE) LCONVERGE(0, ABSOLUTE) PCONVERGE(0.000001, ABSOLUTE)
  /FIXED=Time_years Time_years*Time_years sosvenb0 | SSTYPE(3)
  /METHOD=ML
  /PRINT=SOLUTION TESTCOV
  /RANDOM=INTERCEPT | SUBJECT(id) COVTYPE(VC).

MIXED Apathy_development WITH Time_years sosvenb0
  /CRITERIA=CIN(95) MXITER(100) MXSTEP(10) SCORING(1) SINGULAR(0.000000000001) HCONVERGE(0,
    ABSOLUTE) LCONVERGE(0, ABSOLUTE) PCONVERGE(0.000001, ABSOLUTE)
  /FIXED=Time_years Time_years*Time_years sosvenb0 Time_years*sosvenb0 | SSTYPE(3)
  /METHOD=ML
  /PRINT=SOLUTION TESTCOV
  /RANDOM=INTERCEPT | SUBJECT(id) COVTYPE(VC).

MIXED Apathy_development WITH Time_years skoleb0
  /CRITERIA=CIN(95) MXITER(100) MXSTEP(10) SCORING(1) SINGULAR(0.000000000001) HCONVERGE(0,
    ABSOLUTE) LCONVERGE(0, ABSOLUTE) PCONVERGE(0.000001, ABSOLUTE)
  /FIXED=Time_years Time_years*Time_years skoleb0 | SSTYPE(3)
  /METHOD=ML
  /PRINT=SOLUTION TESTCOV
  /RANDOM=INTERCEPT | SUBJECT(id) COVTYPE(VC).

MIXED Apathy_development WITH Time_years skoleb0
  /CRITERIA=CIN(95) MXITER(100) MXSTEP(10) SCORING(1) SINGULAR(0.000000000001) HCONVERGE(0,
```

```
   ABSOLUTE) LCONVERGE(0, ABSOLUTE) PCONVERGE(0.000001, ABSOLUTE)
  /FIXED=Time_years Time_years*Time_years skoleb0 Time_years*skoleb0 | SSTYPE(3)
  /METHOD=ML
  /PRINT=SOLUTION TESTCOV
  /RANDOM=INTERCEPT | SUBJECT(id) COVTYPE(VC).

MIXED Apathy_development WITH Time_years Psyk_AAO
  /CRITERIA=CIN(95) MXITER(100) MXSTEP(10) SCORING(1) SINGULAR(0.000000000001)
HCONVERGE(0,
   ABSOLUTE) LCONVERGE(0, ABSOLUTE) PCONVERGE(0.000001, ABSOLUTE)
  /FIXED=Time_years Time_years*Time_years Psyk_AAO | SSTYPE(3)
  /METHOD=ML
  /PRINT=SOLUTION TESTCOV
  /RANDOM=INTERCEPT | SUBJECT(id) COVTYPE(VC).

MIXED Apathy_development WITH Time_years Psyk_AAO
  /CRITERIA=CIN(95) MXITER(100) MXSTEP(10) SCORING(1) SINGULAR(0.000000000001)
HCONVERGE(0,
   ABSOLUTE) LCONVERGE(0, ABSOLUTE) PCONVERGE(0.000001, ABSOLUTE)
  /FIXED=Time_years Time_years*Time_years Psyk_AAO Time_years*Psyk_AAO | SSTYPE(3)
  /METHOD=ML
  /PRINT=SOLUTION TESTCOV
  /RANDOM=INTERCEPT | SUBJECT(id) COVTYPE(VC).

MIXED Apathy_development WITH Time_years Log10_DUPplus1
  /CRITERIA=CIN(95) MXITER(100) MXSTEP(10) SCORING(1) SINGULAR(0.000000000001)
HCONVERGE(0,
   ABSOLUTE) LCONVERGE(0, ABSOLUTE) PCONVERGE(0.000001, ABSOLUTE)
  /FIXED=Time_years Time_years*Time_years Log10_DUPplus1 | SSTYPE(3)
  /METHOD=ML
  /PRINT=SOLUTION TESTCOV
```

```
  /RANDOM=INTERCEPT | SUBJECT(id) COVTYPE(VC).


MIXED Apathy_development WITH Time_years Log10_DUPplus1

  /CRITERIA=CIN(95) MXITER(100) MXSTEP(10) SCORING(1) SINGULAR(0.000000000001) HCONVERGE(0,

    ABSOLUTE) LCONVERGE(0, ABSOLUTE) PCONVERGE(0.000001, ABSOLUTE)

  /FIXED=Time_years Time_years*Time_years Log10_DUPplus1 Time_years*Log10_DUPplus1 | SSTYPE(3)

  /METHOD=ML

  /PRINT=SOLUTION TESTCOV

  /RANDOM=INTERCEPT | SUBJECT(id) COVTYPE(VC).


MIXED Apathy_development WITH Time_years Core_SZ

  /CRITERIA=CIN(95) MXITER(100) MXSTEP(10) SCORING(1) SINGULAR(0.000000000001) HCONVERGE(0,

    ABSOLUTE) LCONVERGE(0, ABSOLUTE) PCONVERGE(0.000001, ABSOLUTE)

  /FIXED=Time_years Time_years*Time_years Core_SZ | SSTYPE(3)

  /METHOD=ML

  /PRINT=SOLUTION TESTCOV

  /RANDOM=INTERCEPT | SUBJECT(id) COVTYPE(VC).


MIXED Apathy_development WITH Time_years score_CDSS

  /CRITERIA=CIN(95) MXITER(100) MXSTEP(10) SCORING(1) SINGULAR(0.000000000001) HCONVERGE(0,

    ABSOLUTE) LCONVERGE(0, ABSOLUTE) PCONVERGE(0.000001, ABSOLUTE)

  /FIXED=Time_years Time_years*Time_years score_CDSS | SSTYPE(3)

  /METHOD=ML

  /PRINT=SOLUTION TESTCOV

  /RANDOM=INTERCEPT | SUBJECT(id) COVTYPE(VC).


MIXED Apathy_development WITH Time_years score_CDSS
```

```
  /CRITERIA=CIN(95) MXITER(100) MXSTEP(10) SCORING(1) SINGULAR(0.000000000001)
HCONVERGE(0,
    ABSOLUTE) LCONVERGE(0, ABSOLUTE) PCONVERGE(0.000001, ABSOLUTE)
  /FIXED=Time_years Time_years*Time_years score_CDSS Time_years*score_CDSS | SSTYPE(3)
  /METHOD=ML
  /PRINT=SOLUTION TESTCOV
  /RANDOM=INTERCEPT | SUBJECT(id) COVTYPE(VC).

MIXED Apathy_development WITH Time_years Log10_AUDITplus1
  /CRITERIA=CIN(95) MXITER(100) MXSTEP(10) SCORING(1) SINGULAR(0.000000000001)
HCONVERGE(0,
    ABSOLUTE) LCONVERGE(0, ABSOLUTE) PCONVERGE(0.000001, ABSOLUTE)
  /FIXED=Time_years Time_years*Time_years Log10_AUDITplus1 | SSTYPE(3)
  /METHOD=ML
  /PRINT=SOLUTION TESTCOV
  /RANDOM=INTERCEPT | SUBJECT(id) COVTYPE(VC).

MIXED Apathy_development WITH Time_years AP123Ratio_sum
  /CRITERIA=CIN(95) MXITER(100) MXSTEP(10) SCORING(1) SINGULAR(0.000000000001)
HCONVERGE(0,
    ABSOLUTE) LCONVERGE(0, ABSOLUTE) PCONVERGE(0.000001, ABSOLUTE)
  /FIXED=Time_years Time_years*Time_years AP123Ratio_sum | SSTYPE(3)
  /METHOD=ML
  /PRINT=SOLUTION TESTCOV
  /RANDOM=INTERCEPT | SUBJECT(id) COVTYPE(VC).

MIXED Apathy_development WITH Time_years panss_posfactor_WW_0
  /CRITERIA=CIN(95) MXITER(100) MXSTEP(10) SCORING(1) SINGULAR(0.000000000001)
HCONVERGE(0,
    ABSOLUTE) LCONVERGE(0, ABSOLUTE) PCONVERGE(0.000001, ABSOLUTE)
  /FIXED=Time_years Time_years*Time_years panss_posfactor_WW_0 | SSTYPE(3)
```

/METHOD=ML

/PRINT=SOLUTION TESTCOV

/RANDOM=INTERCEPT | SUBJECT(id) COVTYPE(VC).

MIXED Apathy_development WITH Time_years panss_disfactor_WW_0

/CRITERIA=CIN(95) MXITER(100) MXSTEP(10) SCORING(1) SINGULAR(0.000000000001) HCONVERGE(0,

ABSOLUTE) LCONVERGE(0, ABSOLUTE) PCONVERGE(0.000001, ABSOLUTE)

/FIXED=Time_years Time_years*Time_years panss_disfactor_WW_0 | SSTYPE(3)

/METHOD=ML

/PRINT=SOLUTION TESTCOV

/RANDOM=INTERCEPT | SUBJECT(id) COVTYPE(VC).

****

*3. The prediction model: Introducing independent early predictor variables in order of lifetime appearance, adding interaction effects with time only if the (predictor*time)-interaction was significant in the preceding bivariate association analyses.

*Adjusting for Inclusion Site in the last step.

MIXED Apathy_development WITH Time_years sosvenb0

/CRITERIA=CIN(95) MXITER(100) MXSTEP(10) SCORING(1) SINGULAR(0.000000000001) HCONVERGE(0,

ABSOLUTE) LCONVERGE(0, ABSOLUTE) PCONVERGE(0.000001, ABSOLUTE)

/FIXED=Time_years Time_years*Time_years sosvenb0 | SSTYPE(3)

/METHOD=ML

/PRINT=SOLUTION TESTCOV

/RANDOM=INTERCEPT | SUBJECT(id) COVTYPE(VC).

MIXED Apathy_development WITH Time_years sosvenb0 skoleb0

```
  /CRITERIA=CIN(95) MXITER(100) MXSTEP(10) SCORING(1) SINGULAR(0.000000000001)
HCONVERGE(0,
    ABSOLUTE) LCONVERGE(0, ABSOLUTE) PCONVERGE(0.000001, ABSOLUTE)
  /FIXED=Time_years Time_years*Time_years sosvenb0 skoleb0 | SSTYPE(3)
  /METHOD=ML
  /PRINT=SOLUTION TESTCOV
  /RANDOM=INTERCEPT | SUBJECT(id) COVTYPE(VC).

MIXED Apathy_development WITH Time_years sosvenb0 skoleb0 Psyk_AAO
  /CRITERIA=CIN(95) MXITER(100) MXSTEP(10) SCORING(1) SINGULAR(0.000000000001)
HCONVERGE(0,
    ABSOLUTE) LCONVERGE(0, ABSOLUTE) PCONVERGE(0.000001, ABSOLUTE)
  /FIXED=Time_years Time_years*Time_years sosvenb0 skoleb0 Psyk_AAO | SSTYPE(3)
  /METHOD=ML
  /PRINT=SOLUTION TESTCOV
  /RANDOM=INTERCEPT | SUBJECT(id) COVTYPE(VC).

MIXED Apathy_development WITH Time_years sosvenb0 skoleb0 Log10_DUPplus1
  /CRITERIA=CIN(95) MXITER(100) MXSTEP(10) SCORING(1) SINGULAR(0.000000000001)
HCONVERGE(0,
    ABSOLUTE) LCONVERGE(0, ABSOLUTE) PCONVERGE(0.000001, ABSOLUTE)
  /FIXED=Time_years Time_years*Time_years sosvenb0 skoleb0 Log10_DUPplus1 | SSTYPE(3)
  /METHOD=ML
  /PRINT=SOLUTION TESTCOV
  /RANDOM=INTERCEPT | SUBJECT(id) COVTYPE(VC).

MIXED Apathy_development WITH Time_years sosvenb0 Log10_DUPplus1 score_CDSS
  /CRITERIA=CIN(95) MXITER(100) MXSTEP(10) SCORING(1) SINGULAR(0.000000000001)
HCONVERGE(0,
    ABSOLUTE) LCONVERGE(0, ABSOLUTE) PCONVERGE(0.000001, ABSOLUTE)
  /FIXED=Time_years Time_years*Time_years sosvenb0 Log10_DUPplus1 score_CDSS | SSTYPE(3)
```

```
 /METHOD=ML

 /PRINT=SOLUTION TESTCOV

 /RANDOM=INTERCEPT | SUBJECT(id) COVTYPE(VC).


MIXED Apathy_development WITH Time_years Log10_DUPplus1 score_CDSS

 /CRITERIA=CIN(95) MXITER(100) MXSTEP(10) SCORING(1) SINGULAR(0.000000000001)
HCONVERGE(0,

   ABSOLUTE) LCONVERGE(0, ABSOLUTE) PCONVERGE(0.000001, ABSOLUTE)

 /FIXED=Time_years Time_years*Time_years Log10_DUPplus1 score_CDSS Time_years*score_CDSS
|

   SSTYPE(3)

 /METHOD=ML

 /PRINT=SOLUTION TESTCOV

 /RANDOM=INTERCEPT | SUBJECT(id) COVTYPE(VC).


MIXED Apathy_development WITH Time_years Log10_DUPplus1 score_CDSS
Inclusion_Site_BL_patients

 /CRITERIA=CIN(95) MXITER(100) MXSTEP(10) SCORING(1) SINGULAR(0.000000000001)
HCONVERGE(0,

   ABSOLUTE) LCONVERGE(0, ABSOLUTE) PCONVERGE(0.000001, ABSOLUTE)

 /FIXED=Time_years Time_years*Time_years Log10_DUPplus1 score_CDSS Time_years*score_CDSS

   Inclusion_Site_BL_patients | SSTYPE(3)

 /METHOD=ML

 /PRINT=SOLUTION TESTCOV

 /RANDOM=INTERCEPT | SUBJECT(id) COVTYPE(VC).
```
